# MyeliMetric: A Python-Based Toolbox for Standardized G-Ratio Analysis of Axon-Myelin Integrity

**DOI:** 10.1080/17590914.2025.2603411

**Published:** 2025-12-23

**Authors:** Intakhar Ahmad, Farjana Sultana Chowdhury, Anne I. Boullerne, Alexander Gow, Douglas L. Feinstein

**Affiliations:** a Department of Anesthesiology, University of Illinois, Chicago, Illinois, USA; b Research & Development Service, Jesse Brown VA Medical Center, Chicago, Illinois, USA; c Department of Computer Science, Electrical Engineering and Mathematical Sciences, Western Norway University of Applied Sciences, Bergen (HVL), Norway; d Center for Molecular Medicine and Genetics, Wayne State University School of Medicine, Detroit, MI, USA

**Keywords:** Axon diameter, demyelination, G-ratio analysis, myelin, python toolbox

## Abstract

The g-ratio, defined as the ratio of an axon’s diameter to the total fiber diameter (axon plus myelin), is a key metric for assessing myelin integrity and axonal conduction velocity in both the central and peripheral nervous systems. Deviations from the physiological range often signal underlying pathology. Despite its diagnostic importance, there is currently no standardized, open-source tool for g-ratio analysis from post-segmented electron microscopy images. To address this gap, we developed MyeliMetric, a Python-based, user-friendly toolbox that streamlines g-ratio data preprocessing and integrates biologically informed validation, requiring minimal statistical expertise to operate without introducing common analytical errors. It is built on the principle that g-ratios exhibit relative consistency across varying axon diameters in healthy conditions. To rigorously assess this relationship, MyeliMetric implements a binning strategy that groups axons into biologically relevant diameter cohorts, enabling the detection of size-dependent deviations in g-ratio distributions. This approach addresses common limitations in conventional analyses, including insufficient sampling, pseudo-replication, and artifacts such as misleading regression slopes. Validation using both synthetic and published datasets from rodent models of demyelination demonstrated the tool’s accuracy, reproducibility, and biological relevance. Synthetic data yielded expected outcomes, and in experimental models, MyeliMetric reliably detected reductions in myelin thickness through g-ratio shifts while minimizing artifacts, thereby providing biologically meaningful insights. It is available on GitHub: https://github.com/Intakhar-Ahmad/NeuroMyelin-G-Ratio-Analysis-Toolkit

## Significance Statement

The g-ratio, defined as the ratio of an axon’s diameter to fiber diameter, provides a measure of axon integrity. Methods to calculate g-ratios across various sized axons, and between groups are necessary to ensure correct data interpretation between experiments and laboratories. Under physiological conditions the g-ratio should remain constant, an assumption not kept when using linear regression to fit g-ratio versus fiber diameter. To address reproducibility and allow inspection of fiber diameter dependent changes, we developed MyeliMetric. Myelimetric utilizes a binning strategy to generate subsets based on fiber size, and then calculates average g-ratios for each subset and a grand g-ratio for the entire dataset. Further analyses can be performed on individual or multiple subsets, or on resulting grand g-ratios.

## Introduction

Myelin sheaths are essential components of the central and peripheral nervous systems, enabling saltatory conduction that accelerates signal transmission along axons while reducing conduction time and metabolic demand (Bechler & Ffrench-Constant, 2014; Nave & Werner, 2014). Proper myelination is fundamental to cognitive, sensory, and motor function, as well-myelinated axons transmit action potentials more rapidly and reliably, supporting core neural processes (Fields, 2008; Stadelmann et al., 2019). A key quantitative parameter for assessing myelin integrity is the g-ratio, defined as the ratio of the inner axonal diameter to the total outer fiber diameter (axon plus myelin), originally proposed to optimize conduction velocity (Rushton, 1951) and later adapted for in vivo imaging applications using MRI techniques (Campbell et al., 2018).

In mammals, optimal g-ratios typically fall between 0.6 and 0.8, reflecting a physiological balance between conduction velocity, structural constraints, and energetic efficiency (Chomiak & Hu, 2009; Rushton, 1951; Stikov et al., 2015). Deviations from this range are observed in demyelinating conditions such as multiple sclerosis (MS), hereditary neuropathies like Charcot-Marie-Tooth disease, leukodystrophies such as Pelizaeus-Merzbacher disease, in experimental models including experimental autoimmune encephalomyelitis (EAE) and cuprizone-induced demyelination (Dupree et al., 2015; Nave & Werner, 2014; Stadelmann et al., 2019). These shifts often signal impaired neuronal function, emphasizing the need for precise and reproducible g-ratio quantification.

Despite the g-ratio’s diagnostic and research utility, its broader application is hindered by methodological inconsistencies (Gow, 2025). While segmentation tools like AxonDeepSeg (Zaimi et al., 2018) and MyelTracer (Kaiser et al. 2021) delineate axons and myelin from electron or light microscopy images, they lack integrated support for standardized post-segmentation analysis. Without a unified processing pipeline, researchers frequently resort to ad hoc methods such as manual data handling in Excel or custom-written scripts. These practices introduce variability dependent on the user’s skill level, compromise reproducibility, and heighten the risk of analytical errors. As a result, this methodological gap can lead to inconsistent statistical practices and fragmented results across different studies.

A major pitfall in many studies is interpreting results based on analysis of the relationship between g-ratio and axon diameter. However, recent studies by Gow and colleagues (Gow, 2025; Gow et al., 2025) have revisited the importance of analyzing g-ratio data within the context of the axomyelin unit model, a biologically-grounded framework that asserts a linear relationship between axon and fiber diameters, consistent with classical findings from mid-20th-century studies (Rushton, 1951; Schnepp & Schnepp, 1971). This model offers a more physiologically valid and statistically sound basis for interpreting g-ratio data.

Another critical issue in the g-ratio literature is the misapplication of regression slopes in scatterplots as indicators of biological significance. As reported (Gow, 2025), interpretations drawn from those slopes can misrepresent true biological relationships, especially since g-ratios under healthy conditions tend to remain relatively stable across axon diameters within a given tract. Furthermore, the same authors emphasized that many reported correlations are subject to statistical artifacts, resulting from nonuniform data distributions, inappropriate averaging of pseudo-replicate data, and failure to account for the hierarchical structure of biological measurements. These methodological flaws not only distort the interpretation of g-ratio data but can undermine the reliability and reproducibility of comparative analyses across studies.

Minor variations in g-ratio can also be overinterpreted regarding their functional impact. For instance, classical models of axonal conduction predict that increasing the g-ratio from 0.75 to 0.85 in a 1 µm axon results in only about a 5%–10% reduction in conduction velocity (Rushton, 1951; Waxman & Swadlow, 1977). This highlights the importance of interpreting g-ratio changes within a physiological context in different axon populations. Thus, there is a need for standardized data handling practices, such as binning and clustering, which are essential for conducting biologically meaningful analyses. Binning involves grouping axons into predefined diameter intervals and calculating the average g-ratio values within each bin. This method helps stabilize variance across the dataset, thereby reduces heteroscedasticity, facilitates the detection of biologically meaningful patterns, and highlights whether specific subpopulations (e.g., small vs. large axons) are differentially affected by diseases or treatment conditions. In contrast, clustering categorizes data based on biologically relevant factors such as animal ID, experimental group, or anatomical region. This approach preserves the nested structure of the data, mitigates pseudoreplication, and enables proper statistical modeling using ANOVA or mixed-effects methods (Chomiak & Hu, 2009; Gow, 2025; Lazic, 2010).

To overcome these challenges in g-ratio analysis, we developed MyeliMetric, a toolbox that automates and standardizes post-segmentation processing of axon and myelin data. Grounded in the axomyelin unit model, MyeliMetric integrates biologically informed data cleaning, accurate g-ratio computation, and systematic binning. By minimizing analytical bias and enhancing reproducibility, this tool provides a robust and validated framework for high-confidence g-ratio assessment across diverse experimental models and pathological conditions.

## Materials and Methods

### Python Ecosystem

MyeliMetric is developed in Python 3.7, leveraging a suite of well-established libraries for data processing, statistical analysis, and visualization. Core dependencies include Pandas for tabular data manipulation (McKinney, 2010), NumPy for numerical computations (Harris et al., 2020), and SciPy for statistical tests such as the Shapiro-Wilk normality test and confidence interval estimation (Virtanen et al., 2020). For data visualization, Matplotlib (Hunter, 2007) and Seaborn (Waskom, 2021) are employed to generate histograms and scatter plots. Furthermore, OpenPyXL and xlrd facilitate reading and writing of Excel files, ensuring compatibility with common spreadsheet formats. All dependencies used in the development of MyeliMetric toolbox are summarized in Table 1.

**Table 1.** Required python libraries for running the MyeliMetric toolbox.

Package	Purpose
tkinter	GUI framework for building the application window
pandas	Data handling and manipulation (e.g., Excel I/O)
numpy	Numerical operations
matplotlib	Plotting visualizations (e.g., histograms, scatter plots)
scipy	Scientific computations/statistics (used in analysis modules)
openpyxl	Read/write Excel files with pandas
pillow	Image loading for GUI background
diptest	Test for multimodality in a univariate distribution
xlsxwriter	Alternative engine for writing Excel files
statsmodels	It provides classes and functions for the estimation of statistical models

MyeliMetric can be launched directly via the “main.py” script using any standard Python 3.7+ environment. A script named “install_dependencies.py” is provided in the script folder to streamline setup by automatically installing the required Python packages. For users without a Python installation, a portable Windows version with all dependencies pre-configured is also available as a download from the repository.

### Modular Architecture and Data Handling

**Figure 1.**
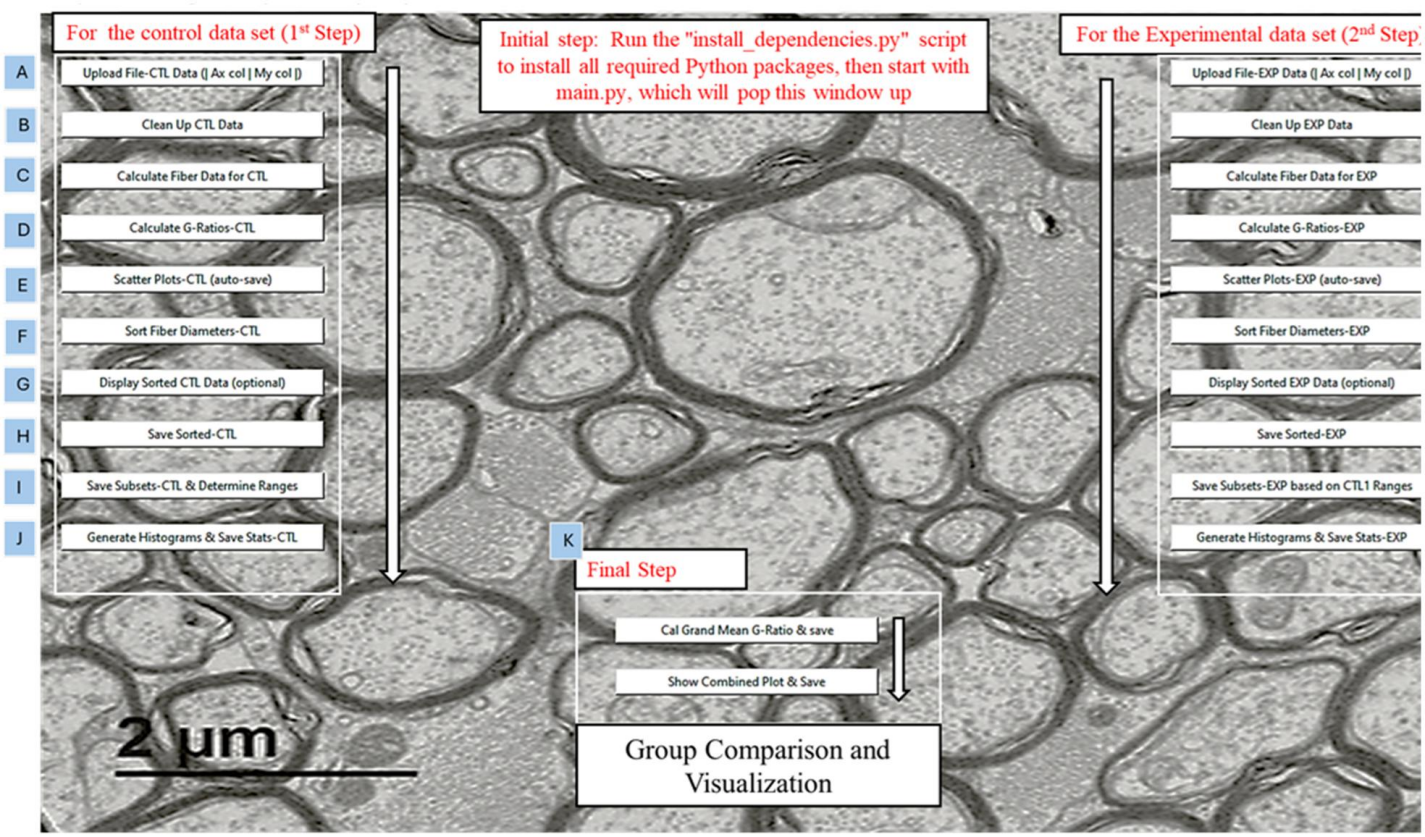
Modular graphical user interface (GUI) of the MyeliMetric toolbox. A screenshot of the opening interface of the MyeliMetric software application. The modular layout supports a stepwise workflow, beginning with dependency installation. The interface is organized into three sequential sections. Users first process the control group (left panel), which includes: (A) data upload, (B) data cleaning, (C) fiber diameter calculation, (D) g-ratio computation, (E) scatter plot generation, (F) data sorting, (G) data display, (H) output saving, (I) subset definition and reference range determination, and (J) histogram generation with statistical annotations. The experimental dataset (right panel) follows the same workflow. In the final step (K, bottom panel), users can calculate the grand mean g-ratio across groups and generate integrated visualizations, enabling comprehensive comparisons of treatment effects on myelin integrity.

MyeliMetric is designed with a modular, offline-first architecture to ensure robustness when handling large datasets and to securely manage sensitive experimental data. This modular user interface (UI) layout (Figure 1) separates each functional component: data import, cleaning, computation, analysis, comparison, and visualization, allowing them to operate independently while functioning cohesively. This structure enhances maintainability, simplifies debugging, and supports future expansion or integration with other analysis pipelines. Additionally, the modularity fosters transparency by clearly delineating the role and boundaries of each processing step, making the tool accessible to both novice and experienced users.

The toolbox begins with the IO Handler module, which reads .xls input files, checks for required column headers (e.g., SampleName_Ax for axon diameter and SampleName_My for myelin thickness), and organizes the contents into internal pandas.DataFrame objects for downstream analysis in Python. For each sample, the column names must share an identical prefix (SampleName) to ensure the correct pairing of axon and myelin data. These input files should contain numerical values in float64 format, representing micrometer-scale measurements. If myelin thickness is provided as a one-sided radial measurement, it must be multiplied by two before analysis. Example input files conforming to this structure are provided in the repository to facilitate correct formatting and usage.

## Analytical Rationale and Workflow

To ensure biologically meaningful g-ratio calculations, in addition to removing missing entries, data cleaning thresholds are applied to exclude axon diameters smaller than 0.15 µm and myelin thicknesses below 0.03 µm. These cutoffs are supported by multiple ultrastructural studies. In 1980, it was reported that axons smaller than 0.15 µm are rare and may lack functional significance (Sturrock, 1980), while Hildebrand and Hahn noted that extremely thin myelin sheaths (<0.03 µm) are unlikely to provide effective insulation (Hildebrand & Hahn, 1978). More recently, it has been demonstrated that in the central nervous system, oligodendrocytes typically myelinate axons larger than 0.3 µm, reinforcing the existence of a lower functional threshold (Arancibia-Cárcamo et al., 2017). Axons smaller than ∼0.2 µm are frequently unmyelinated or inconsistently myelinated, making g-ratio estimates in this range biologically unreliable.

To facilitate stratified analysis of axonal myelination profiles, MyeliMetric implements a binning strategy that partitions axons into six predefined diameter-based subgroups. This framework accounts for the inherent heterogeneity in axon calibers observed across the central nervous system, where axon diameter is a key determinant of myelin sheath thickness and conduction velocity. Grouping axons into discrete size categories enables quantitative comparisons that can uncover diameter-specific patterns of myelin integrity, which may be obscured in bulk or non-stratified analyses. This approach is biologically justified by foundational studies demonstrating that larger axons exhibit greater reliance on myelin for efficient signal conduction and are differentially susceptible to demyelination (Hildebrand & Hahn, 1978; Waxman & Bennett, 1972). The use of six bins reflects an optimal compromise between analytical resolution and statistical power. Fewer bins (e.g., 2–3) may oversimplify the data and obscure subgroup-specific trends, while more bins (e.g., 10 or more) may result in sparse subgroups, reducing the robustness of statistical comparisons. Theoretically, six bins provide sufficient granularity for interpretability while maintaining adequate sample sizes within each category (Gow, 2025; Gow et al., 2025). This binning strategy aligns with the broader analytical approaches used in previous ultrastructural and MRI-based g-ratio studies, which emphasize the importance of examining g-ratio variation as a function of axon caliber (Arancibia-Cárcamo et al., 2017; Stikov et al., 2015). The range boundaries for the six subgroups are derived from the distribution of axon diameters in the control dataset, which provides a representative baseline for biologically plausible values. These bin ranges are then applied consistently across all experimental groups to support standardized comparisons and maintain interpretive consistency throughout the analysis.

## Simplified Pseudocode

### G-Ratio Analysis

BEGIN

LOAD input Excel files (e.g., control or experimental group)

VERIFY required columns (e.g., SampleName_Ax and SampleName_My)

CLEAN data:

REMOVE entries with missing values

APPLY thresholds:

- EXCLUDE axon diameter < 0.15 µm

- EXCLUDE myelin thickness < 0.03 µm

LOG all exclusions and modifications

CALCULATE fiber diameter = axon diameter + myelin thickness

CALCULATE g-ratio = axon diameter/fiber diameter

BIN axons into 6 predefined diameter categories:

- Bin ranges derived from the control dataset (CTL1) distribution

- APPLY the same binning ranges to all groups for consistency

FOR each bin:

CALCULATE mean, median, and standard deviation

PERFORM normality test (i.e., Shapiro-Wilk)

GENERATE histograms and scatter plots

RETURN summary statistics and visual outputs

END

### G-Ratio Comparison

BEGIN

LOAD data for control and experimental groups

VALIDATE presence of required columns: axon_diameter, myelin_thickness

CLEAN both datasets (remove missing or invalid entries)

IF both control AND experimental datasets are available:

FOR each matching bin:

PERFORM statistical comparison (i.e., the 2-way ANOVA)

# Comparative statistical analysis can be performed on the output as needed, depending on the experimental design.

GENERATE comparative plots:

- Histograms of g-ratios

- Scatter plots of g-ratio vs axon diameter

SAVE outputs (plots, mean ± SD, and cleaned data) to the specified output directory

END

**Figure 2.**
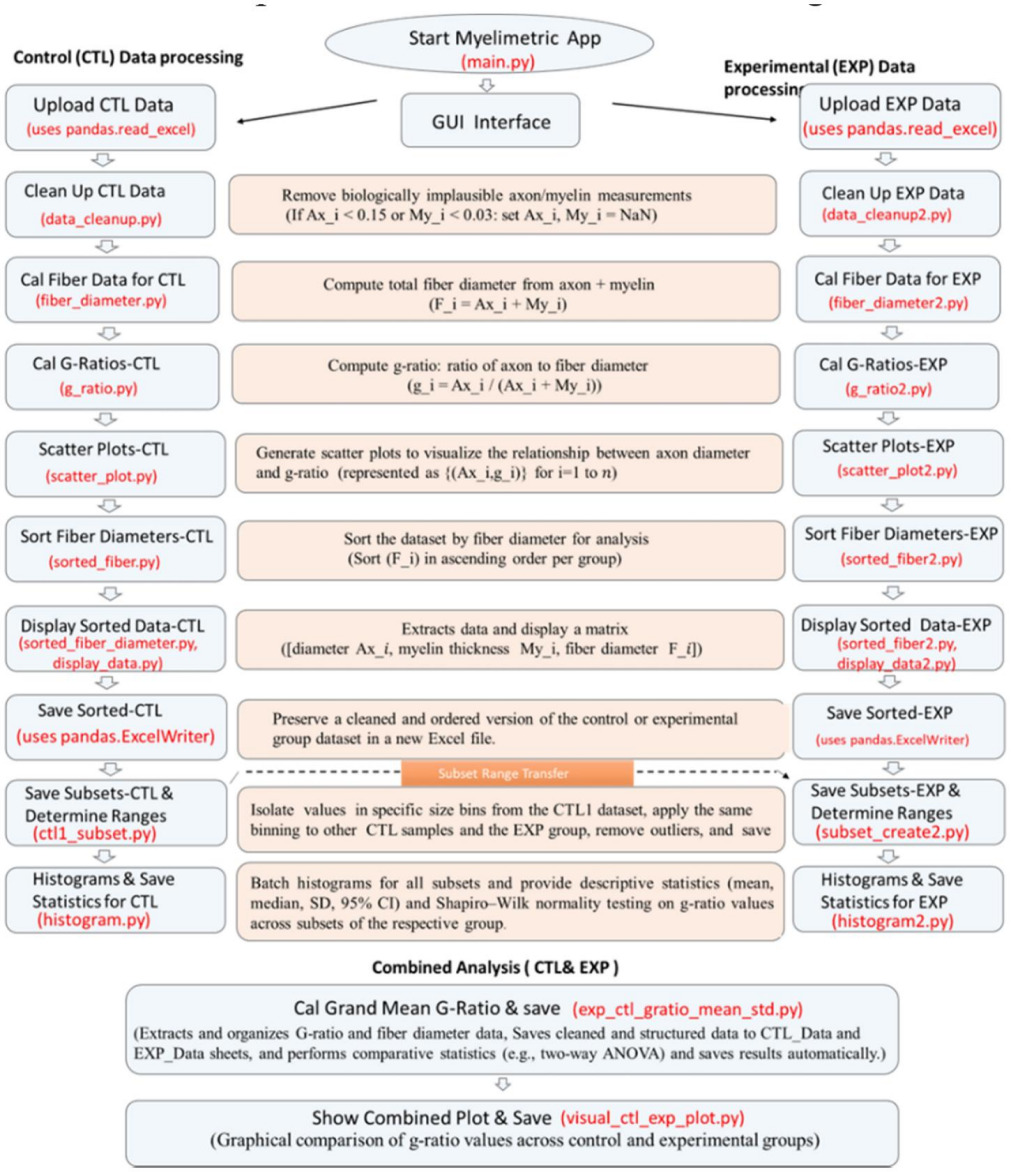
Architecture of the MyeliMetric toolbox. The MyeliMetric pipeline consists of distinct but interconnected scripts organized into functional modules for processing control (CTL) and experimental (EXP) datasets. Each module performs a specific analytical task, including data cleaning, fiber diameter calculation, g-ratio computation, scatter plotting, data sorting, and statistical evaluation. Buttons in the GUI are annotated with their corresponding implementing scripts, providing a transparent and traceable workflow.

To enhance transparency and reproducibility, the modular architecture of MyeliMetric is detailed in Figure 2, which outlines the scripts, their core functions, operational formulas, and specific roles within the analysis pipeline. This structured breakdown enables users to understand the internal logic of the software and facilitates the selective execution of individual components as needed. A full user’s guide is available as supplemental data.

## Evaluation Plan

MyeliMetric was validated through a three-tier process to ensure reliability and performance. In the first tier, unit tests were performed on custom-developed modules that are not part of any existing Python libraries. These tests were used to simulate edge cases such as missing columns, extreme values, and inconsistent data formats. All operations were systematically logged to ensure full traceability and reproducibility of the results. In the second tier, a large synthetic dataset was generated by mimicking the statistical properties of real experimental data, allowing performance benchmarking under realistic conditions. In the third tier, the software was applied to published demyelination datasets to assess its ability to reproduce known g-ratio patterns. All unit test scripts are provided in the repository to ensure reproducibility and transparency.

## Synthetic Data Generation Reflecting Real Experimental Properties

To evaluate the accuracy and robustness of g-ratio computations under controlled conditions, we generated synthetic datasets that mirror real axon and myelin measurements (Dupree et al., 2022). We created control (CTL) and cuprizone-treated experimental (CPZ) conditions, each with five individual animals per group (CTL1 – CTL5, CPZ1– CPZ5) containing n = 1,000 baseline axons per subgroup. Axon diameters (a, in µm) were sampled from a log-normal distribution in log space (μ = 1.0, σ = 0.5) to cover the empirical interquartile range of ∼0.3–1.2 µm observed in our reference dataset, providing a biologically plausible spectrum while permitting a realistic right tail of larger fibers. For each axon, the g-ratio (g) was drawn from a truncated normal distribution N(g̅, 0.03^2^) bounded to [0.65, 0.95], with condition-specific means of g̅ = 0.7 for CTL and g̅ = 0.8 for CPZ. To introduce biological replicate structure, we partitioned each condition into five subgroups with subgroup-specific means μg = μg + δ, where δ ∈ {−0.02, −0.01, 0, +0.01, +0.02}. Rather than sampling myelin thickness independently, we enforced the g-ratio identity g = a/(a + 2 m) and solved deterministically for myelin thickness as m = a × (1/g - 1)/2, ensuring every observation has a mathematically exact g-ratio, with fiber diameter computed as f = a + 2 m. The base ratio r = m/a = 0.5 × (1/g - 1) determines the fiber-to-axon scaling f/a = 1 + 2r, yielding mean values of r̅ ≈ 0.141 (f/a ≈ 1.282) for CTL and r̅ ≈ 0.095 (f/a ≈ 1.190) for CPZ, with approximate 5th–95th percentile ranges of 0.10–0.18 for CTL and 0.06–0.13 for CPZ given the simulated spread (SD ≈ 0.03). To maintain rodent CNS plausibility, we applied a fiber cap via rejection sampling, accepting draws only if f ≤ 2.0 µm. For sensitivity analyses, we appended 100 extreme rows per subgroup representing rare small-caliber fibers with very thin myelin by sampling aext ∼ Uniform(0.05, 0.149) µm and m_ext ∼ Uniform (0.005, 0.029) µm, guaranteeing a_ext < 0.15 µm and m_ext < 0.03 µm, with corresponding g_ext = a_ext/(a_ext + 2 m_ext) and f_ext = a_ext + 2 m_ext recalculated and the fiber cap reapplied. Quality control removed any non-finite or non-positive values, and random seeds were fixed to ensure reproducibility. The final structure comprised 5,500 rows per condition (five subgroups × 1,100 rows each), providing internally consistent observations with known ground-truth g-ratios and realistic fiber distributions bounded by biological limits. For analysis, g-ratios were computed at three levels: individual axon gi = ai/(ai + 2 mi), subgroup mean g subgroup = (1/n) × Σgi, and condition mean (g̅) = Σg subgroups. When fed into analytical applications, datasets were labeled as CTL for control and EXP for experimental/treatment conditions.

## Results

### Validation

Unit tests were conducted on all custom-developed modules to validate their functional correctness and robustness. The results confirmed that each function executed as intended, produced accurate and consistent outputs, and effectively handled atypical input scenarios, including edge cases. No functional errors or unexpected behaviors were detected during testing. A comprehensive summary of the tested modules, test scope, outcomes, and associated external dependencies is presented in Table 2.

**Table 2.** Verification of core functions in the MyeliMetric toolbox via unit testing (external module dependencies).

Unit test script	Module to be tested	Tested	Status
test_data_cleanup.py	data_cleanup.py data_cleanup2.py	Verifies filtering based on thresholds (axon < 0.15, myelin < 0.03) and realignment of non-NaN values	Pass
test_subset.py	ctl1_subset.py	Functions related to subset creation and range saving Test subset creation and min/max validation Test filtering and correct sheet naming Test saving range data and removing empty sheets	Pass
test_g_ratio.py	g_ratio.py g_ratio2.py	Correctness of g-ratio values (Ax / (Ax + My), rounded to 2 decimals) and verifies input columns are present and expected output columns are created	Pass

### Validation against Synthetic Data

**Figure 3.**
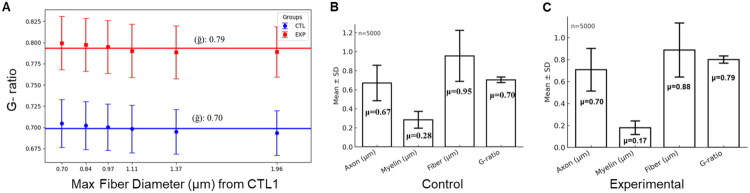
Validation of MyeliMetric application functionally using a synthetic dataset. (A) Pipeline for dataset construction: axon diameters were sampled from a log-normal distribution to match biological size spectra; g-ratios were drawn from a truncated normal (CTL, mean ≈ 0.70) and CPZ-treated (EXP, mean ≈ 0.79) with small subgroup offsets. Stratification into six fiber-diameter bins preserved the size distribution and revealed stable within-condition mean g-ratios with a consistent CTL < EXP separation across bins. (B-C) Pooled distributions reproduced the intended group difference: EXP showed a higher g-ratio (thinner myelin relative to axon) than CTL, consistent with the imposed means.

The application output confirmed that the synthetic datasets accurately reproduced the targeted g-ratio distributions, with control (CTL) groups converging to a mean g-ratio of 0.70 and experimental (EXP) groups converging to 0.79, closely matching the imposed parameters and reflecting expected differences in myelination status where higher g-ratios indicate reduced myelin thickness relative to axon diameter (Figure 3A). The stratification revealed consistent g-ratio differences between CTL and EXP conditions across all 6 diameter bins, validating the uniformity of the simulated treatment effect. In the pooled datasets, CTL and EXP confirm the intended group difference, with EXP exhibiting reduced myelin investment and a higher g-ratio compared to CTL (Figure 3B,C). Robustness testing through sensitivity analysis, which appended 100 extreme observations per subgroup (axon diameter <0.15 µm, myelin thickness < 0.03 µm), demonstrated that these outliers were appropriately excluded by the quality control algorithms, with mean g-ratio calculations remaining stable (ΔCTL < 0.017, ΔEXP < 0.014) when extreme values were included versus excluded, indicating that the analytical workflow is robust to rare small-caliber, thin-myelin outliers that may arise from tissue preparation artifacts or segmentation errors, thus confirming the methods’ suitability for real-world datasets where such outliers occasionally occur.

### Validation against Published Data

**Figure 4.**
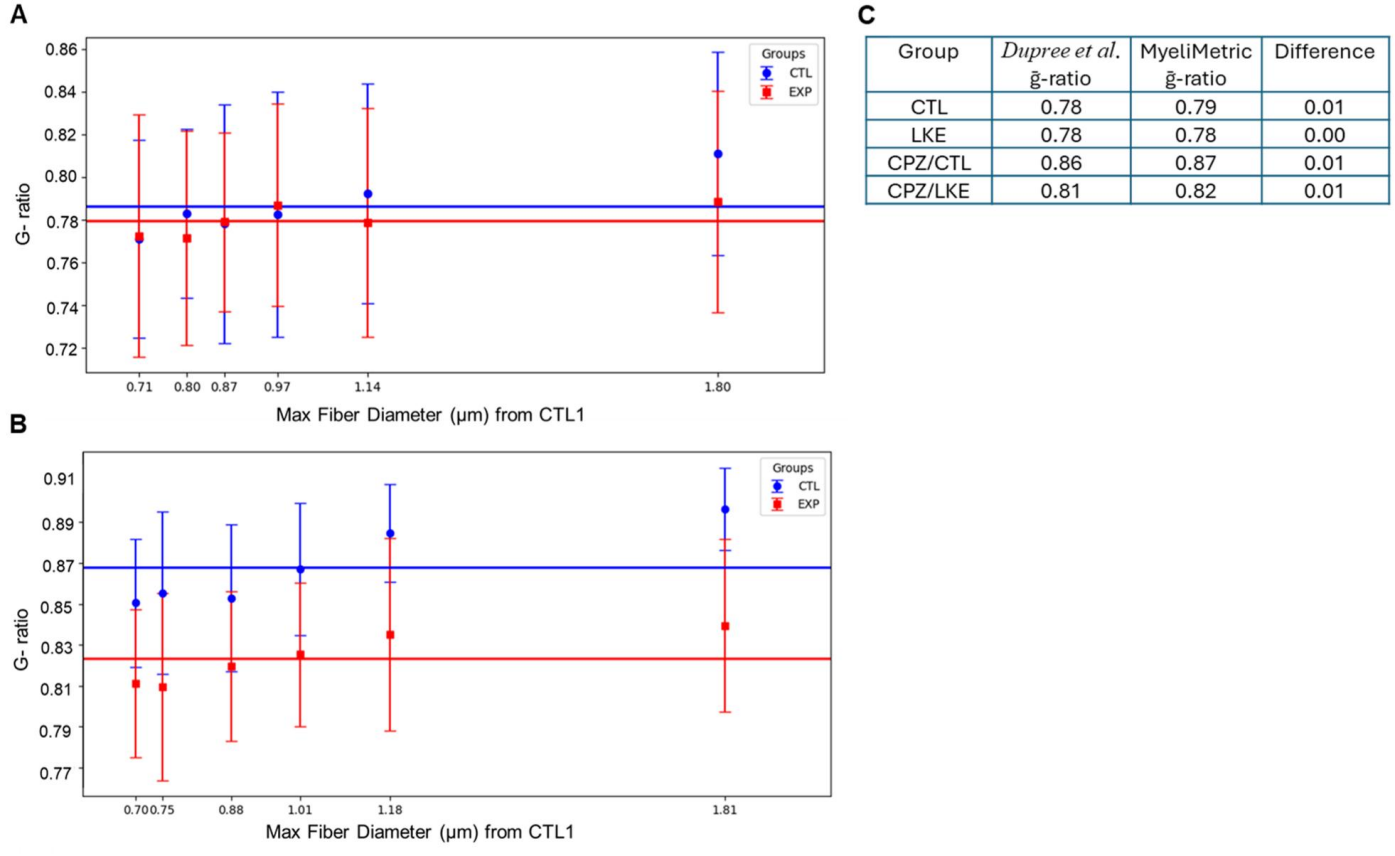
Validation of MyeliMetric using a published dataset. The mean g-ratios for each of the 6 fiber-diameter bins were calculated using MyeliMetric for (A) Control (CTL) and LKE-treated (EXP) groups and (B) cuprizone-treated mice followed by either control chow (CTL) or LKE chow (EXP). Grand mean g-ratios (g̅) were obtained by fitting the constant-g model to the six bin means. (C) The computed grand mean g-ratios compared to those reported by Dupree et al. (2022).

To test MyeliMetric against real-world experimental data, we re-analyzed a published dataset from a study examining cuprizone-induced demyelination in mice with and without Lanthionine Ketimine Ethyl Ester (LKE) supplementation (Dupree et al., 2022). For the CTL and LKE-only treated groups (Figure 4A), the mean g-ratios for each subset were similar; as were the calculated grand mean g-ratios (g̅) which closely matched the originally reported values (Figure 4C). Similarly, the g̅ values calculated by MyeliMetric for recovery with and without LKE (Figure 4B) closely match the values reported in the original study. Minute deviations were observed, as expected, mainly due to differences in outlier exclusion, binning strategies, and data handling protocols used by MyeliMetric, which implements stricter filtering criteria for physiological plausibility.

**Figure 5.**
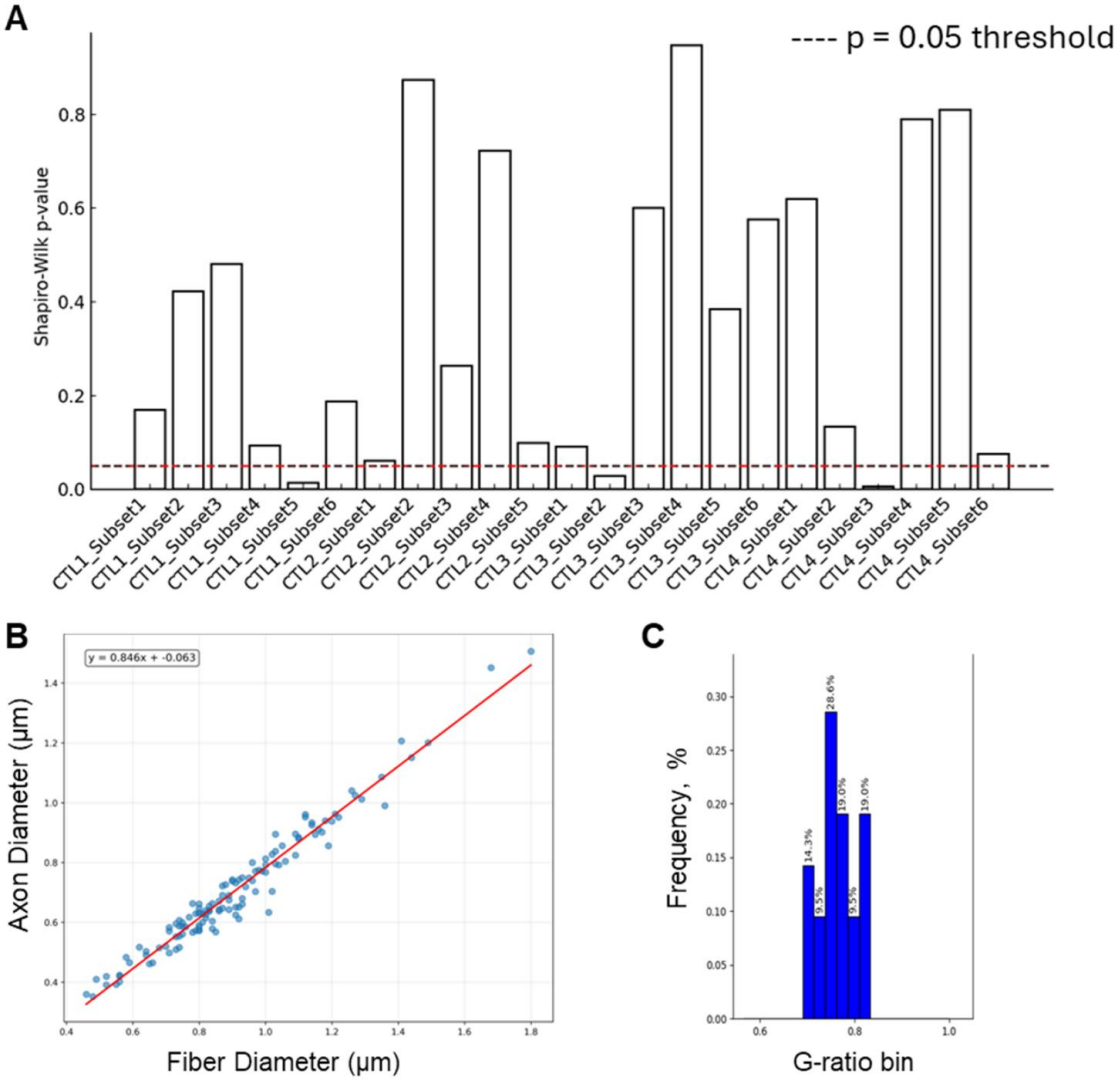
Statistical and visual profiling of diameter-binned g-ratio data generated by MyeliMetric. (A) Shapiro–Wilk normality testing across fiber diameter subsets generated from the control (CTL) dataset identified deviations from Gaussian distribution in three bins (*p* < 0.05, red line), highlighting data heterogeneity. MyeliMetric-generated (B) Scatter plot of axon diameter versus fiber diameter (µm) with a linear fit, illustrating the expected axon–fiber relationship, and (C) Subject-level g-ratio histogram (frequency, %) visualizing the within-animal distribution underlying the bin-wise means.

Importantly, MyeliMetric introduced enhanced analytical granularity by segmenting axons into diameter-based bins, revealing fiber-size-specific responses to LKE treatment. For instance, while the original study reported a significant reduction in g̅-ratio following LKE administration after two weeks of remyelination (CPZ/LKE vs. CPZ/CTL), a 2-way ANOVA analysis on data generated by MyeliMetric demonstrated that the Group × Subset (fiber diameter bins) interaction approached but did not reach statistical significance (*F* (5, 994) = 1.99, *p* = 0.078). This implies that while both group and subset independently influence g-ratio values, there is no strong evidence that the effect of group differs systematically across fiber diameter subsets. Additionally, Shapiro–Wilk normality testing in subgroups revealed deviations from Gaussian distribution in some bins, although the low standard errors inherent in this test could increase the number of false-positives. This statistical profiling gives an overview of data structure and variability (Figure 5A). MyeliMetric also generated scatter plots of fiber diameter versus axon diameter for individual samples, alongside subset-specific histograms for each subject (Figure 5B), providing a detailed visualization of axon–myelin relationships. These plots facilitate a deeper understanding of the distributional properties within and across fiber diameter subsets, thereby enhancing the interpretability at both the individual and subset (bin) levels.

## Discussion

MyeliMetric advances the current landscape of g-ratio analysis; it is a purpose-built Python toolbox that enhances the post-segmentation analysis of axon–myelin structures by implementing biologically grounded and statistically rigorous workflows. Rather than relying on fragmented or ad hoc approaches, MyeliMetric offers an integrated solution that improves the reliability of g-ratio estimation through standardized data processing and contextual validation. Its performance demonstrated across both synthetic and experimental datasets highlights its utility in uncovering subtle, fiber-size-dependent patterns in myelin architecture, an advancement over conventional methods that often overlook such granularity.

Enhanced resolution in g-ratio analysis is particularly important in demyelinating conditions such as multiple sclerosis (MS), where remyelination dynamics can vary significantly across axon calibers. Previous studies have shown that large-caliber axons tend to exhibit greater spontaneous remyelination capacity during recovery (Franklin & Goldman, 2015), highlighting the importance of size-specific assessment. This pattern reinforces the need for granular post-segmentation analysis to accurately evaluate treatment effects. Notably, such selective remyelination aligns with findings of enriched populations of specific remyelinating oligodendrocytes, such as ermin-expressing cells within active repair zones of MS lesions (Ahmad et al., 2021). These observations collectively emphasize that therapeutic responses are not uniformly distributed across all axons, and tools like MyeliMetric are essential for detecting and interpreting such biologically relevant heterogeneity.

Given that control datasets serve as the physiological reference frame it is both reasonable and necessary to apply biologically informed filters during analysis. The inclusion of implausible values, such as those resulting from segmentation artifacts or imaging inconsistencies that can distort statistical measures and lead to misleading subgroup classifications. In contrast, experimental datasets may legitimately contain atypical values that reflect pathological or regenerative changes, including severely demyelinated axons or unusually thin myelin sheaths. To account for this distinction, MyeliMetric incorporates a flexible filtering system that allows users to enable or bypass the data cleanup module based on the biological context. Its modular design supports this adaptability while maintaining analytical integrity. Furthermore, all excluded data are automatically logged in a separate output file, promoting transparency and enabling users to review, audit, or reprocess the filtered entries as needed.

Another crucial advantage in analysis with the MyeliMetric application is its integration of biological plausibility checks. The software flags unexpected correlations between fiber diameter and g-ratio, prompting users to investigate potential methodological artifacts such as segmentation bias, inconsistent imaging resolution, or sampling error (Chomiak & Hu, 2009). Additionally, g-ratio values falling outside of the expected physiological range (typically <0.5 or >0.9) are automatically removed as potential outliers. These built-in quality control features are essential for ensuring scientific rigor and reproducibility, particularly in multicenter or longitudinal studies where methodological variability can be a significant confounding factor.

Simulated datasets modeled after empirical axon and myelin measurements offered a controlled environment to evaluate the reliability of MyeliMetric under physiologically realistic conditions. The tool consistently returned g-ratio values within the expected range while filtering out entries that lacked biological plausibility. This performance is particularly important given the narrow window within which g-ratios are considered physiologically normal.

A re-analysis of experimental data from a cuprizone-induced demyelination model (Dupree et al., 2022) further highlights the utility of MyeliMetric. While the original study reported a significant reduction in g-ratio following LKE treatment, our stratified analysis revealed a more nuanced effect, with group × subset interactions (based on fiber diameter bins) approaching statistical significance. This finding underscores the value of bin-wise comparisons and mixed-model approaches, particularly in studies with moderate sample sizes or heterogeneous axonal populations (Gow et al., 2025; Lazic, 2010). Additionally, the identification of non-Gaussian distributions in several diameter bins reinforces the need to incorporate normality testing as a routine step in g-ratio analyses, an aspect often neglected in previous work.

While tools such as AxonDeepSeg (Zaimi et al., 2018) and MyelTracer (Kaiser et al., 2021) provide high-quality segmentation of axons and myelin from microscopy images, they do not offer integrated post-processing pipelines for g-ratio computation, outlier filtering, or biologically contextualized analysis. MyeliMetric fills this critical gap by providing a modular, transparent framework that enhances reproducibility and reduces user-induced variability. Designed to complement existing segmentation platforms, MyeliMetric streamlines post-segmentation workflows and supports downstream export to statistical environments such as R, Python’s statsmodels, or machine learning frameworks. This interoperability is especially important as neuroscience increasingly embraces integrated, multi-modal analyses. By enabling high-confidence morphological quantification, MyeliMetric supports the alignment of imaging-based findings with transcriptomic, proteomic, and behavioral data, facilitating deeper insights into both systems neuroscience and translational research.

Despite its strengths, MyeliMetric has several limitations. First, the toolbox relies on the accuracy of upstream segmentation and cannot validate or correct errors introduced during image preprocessing or axon/myelin labeling (Zaimi et al., 2018). As a result, it may not adequately account for irregularly shaped or obliquely sectioned axons, which can distort area-to-diameter conversions. Second, while the tool is optimized for high-resolution electron microscopy data, its performance on lower-resolution or noisy datasets, such as those generated by light microscopy requires further validation. Additionally, the current version supports only .xlsx file formats, limiting compatibility with alternative data structures commonly used in large-scale or automated pipelines. At present, advanced statistical analyses must be performed externally, as they are not integrated into the software. Future iterations may include 3D volumetric support, machine learning–based outlier detection, expanded file format compatibility, and embedded statistical comparison modules to enhance both scalability and user accessibility.

In summary, MyeliMetric addresses a critical methodological need in neurobiological research by offering a validated, reproducible, and user-friendly pipeline for g-ratio analysis. It represents a significant advancement in g-ratio quantification by providing a biologically grounded, quality-controlled, and statistically robust analysis pipeline. Its ability to resolve fiber-size-specific patterns, detect artifacts, and support reproducible workflows addresses long-standing challenges in axon–myelin research. As experimental neuroscience continues to evolve toward higher-resolution, multimodal investigations, MyeliMetric offers a foundational tool for high-confidence morphometric analysis in both basic and translational myelin research.
